# Why do employees commit fraud? Theory, measurement, and validation

**DOI:** 10.3389/fpsyg.2022.1026519

**Published:** 2022-10-06

**Authors:** Bin Lin, Junqin Huang, Youliang Liao, Shanmin Liu, Haiyan Zhou

**Affiliations:** ^1^School of Business, Sun Yat-sen University, Guangzhou, China; ^2^Big Data Auditing and Governance Lab, Guangdong Industry Polytechnic, Guangzhou, China; ^3^Foshan State-Owned Assets Supervision and Administration Commission, Foshan, China; ^4^School of Economics and Management, South China Normal University, Guangzhou, China; ^5^Robert C. Vackar College of Business and Entrepreneurship, University of Texas Rio Grande Valley, Edinburg, TX, United States

**Keywords:** employee, fraud motive, scale development, theory of planned behavior, unethical behavior, white-collar crime

## Abstract

Previous research on corporate governance has extensively explored the motives of corporate fraud. However, this research has paid little attention to employees, the real executors of fraud, resulting in the psychological and behavioral decision-making process of employees who commit fraud in enterprises becoming a “black box” that has not yet been opened. Based on the theory of planned behavior, our study integrates the existing research findings on driving factors of employee fraud and anti-fraud practical experience, extracts the key factors of employee fraud motive, and develops a multidimensional scale of employee fraud motive. The exploratory factor analysis (EFA) generates three subscales, comprising 14 items, measuring attitude, subjective norm and perceived behavioral control of employee fraud motive. The confirmatory factor analysis (CFA) supports the reliability, discriminant validity and convergent validity of the new scale. The multiple regression results show that the score of employee fraud motive is positively correlated with the amount of employee fraud occurrence, indicating that the predictive validity of the scale holds. Overall, the scale developed in our study displays good reliability and validity, and is worth spreading.

## Introduction

Fraud is a persistent problem in corporate governance and is increasingly becoming a global focus. According to *Occupational Fraud 2022*, published by the Association of Certified Fraud Examiners (ACFE), the economic loss from fraud in all types of organizations, including governments and businesses, is about 5% of their total annual revenue, with an average loss of $1,783,000 per case. The former UN Secretary-General Ban Ki-moon characterized corruption as a “global threat” on the same level as terrorism and climate change. The empirical research has found that fraud causes the firm to suffer direct losses and penalty losses, leads to serious damage to reputation, and adversely affects both the capital and product markets of the firm (Amiram et al., 2018).

Employees are the executors of corporate fraud. Regardless of the form and purpose, corporate fraud is ultimately carried out by individuals to serve the interests of individuals. The individuals here are the employees of the enterprise, including management and general employees. The classical definition of fraud also reflects that employees are the executors of corporate fraud. ACFE defines fraud as the use of one’s occupation for personal enrichment through the deliberate misuse or misapplication of the employing organization’s resources or assets. The Institute of Internal Auditors (IIA) defines fraud as an act in which the perpetrator intentionally deceives and damages others for his or her own personal benefit. Employees are the executors of corporate fraud, meaning that every employee has the potential to become a fraudster. The study of the motives of employee fraud plays a vital role in identifying potential perpetrators, blocking potential perpetrators from evolving into real perpetrators, and preventing the occurrence of corporate fraud (Dorminey et al., 2012).

However, the literature in the field of corporate governance have paid insufficient attention to employees when studying corporate fraud, considering firms as the executors of fraud. First, limited by the availability of data, most current research still defines fraud at the firm level and uses firm-level data to measure fraud [e.g., litigation, restatements, enforcement announcements (Karpoff et al., 2017)] and focuses primarily on financial or accounting fraud (Hogan et al., 2008), yet such definitions represent only a small portion of corporate fraud. ACFE classifies fraud into three major categories: corruption, asset misappropriation, and financial statement fraud. While according to *Occupational Fraud 2022*, 86% of fraud case types are asset misappropriation and only 9% of financial statement fraud. Second, correspondingly, current research has limited the search for the antecedents of fraud to firm-level factors (Dechow et al., 2011; Perols et al., 2017; Bao et al., 2020; Xu et al., 2022), paying insufficient attention to the individual-level factors, the factors that account for larger percentage in the variance of fraud losses (Holtfreter, 2008; Timofeyev, 2015). While in the field of similar research, white-collar crime and unethical behavior, the literature focuses more on influencing factors at the individual level. According to a meta-analysis from Pusch and Holtfreter (2021), the number of individual predictors of white-collar crime account for 76%, and the number of organizational predictors account for only 24%. In a review of unethical behavior, Trevino et al. (2014) devote a great deal of space to reviewing the individual-level factors of unethical behavior. In conclusion, the lack of emphasis on employees in corporate governance research has resulted in that the psychological and behavioral decision-making process of employees who fraud in enterprises has been a “black box” that has not yet been opened.

The research findings on unethical behavior and white-collar crime and experience working in anti-fraud practice provide a rich set of individual-level motives for fraud. The literature in the area of unethical behavior and white-collar crime delve into the motives of fraud at the individual level, finding partial psychological variables associated with fraud, such as job satisfaction (Dalal, 2005; Judge et al., 2006; Zhang, 2020), Machiavellian personality (Hegarty and Sims, 1978; Castille et al., 2018; Manara et al., 2020), self-control (Hirschi and Gottfredson, 1987; Gino et al., 2011; Joosten et al., 2014), and so on. Individual-level motives of fraud have also been summarized in anti-fraud practice experience. For example, a well-known anti-fraud practice experience is the U.S. *Statement on Auditing Standards* No. 99, *Fraud Risk Factors and Content* (SAS No. 99; hereinafter referred to as “*Fraud Auditing Standards*”). The *Fraud Auditing Standard* uses the fraud triangle theory, which considers the three dimensions of fraud motive, including pressure, opportunity, and rationalization, and lists the precursor manifestations of perpetrators under each dimension. However, the employee fraud motives in these findings have not yet been integrated, resulting that the question of which of the many fraud motives are most important remain unanswered.

Therefore, our study considers employees as the executors of corporate fraud, summarizes the motives of fraud at the employee level, and develops the employee fraud motive scale. In the scale development process, we form the initial scale based on the theory of planned behavior, integrating the research findings of unethical behavior and white-collar crime as well as the experience of anti-fraud practice work.

Specifically, we first integrate the research findings of unethical behavior and white-collar crime as well as the experience of anti-fraud practice work to form the initial employee fraud motive scale. Then, using questionnaire data from corporate anti-fraud leaders, we implement exploratory factor analysis (EFA) and confirmatory factor analysis (CFA) based reliability and validity tests to extract the key factors of employee fraud motive and develop the final scale. The final scale includes three subscales of attitudes, perceived behavioral control, and subjective norm, with 14 items. Finally, we use the questionnaire data of the final scale to verify the predictive validity of the scale and to analyze the internal structure of the employee fraud motive.

The main contributions of our research are as follows. Firstly, based on the behavioral perspective, we summarize the fraud motives at the employee level and develop the employee fraud motive scale, enriching the research on corporate fraud motives. Secondly, our study theoretically and quantitatively integrates the existing research findings and practice experience to identify the key factors of fraud motive, which not only implicate researchers to focus on the important issues, but also help anti-fraud practitioners to save costs. Thirdly, our research comprehensively and directly measures the employee fraud motive by developing a scale, providing a quantitative basis for empirical research on employee fraud motive. Finally, our study provides an operational tool for corporate anti-fraud practitioners to diagnose the causes of fraud and to prevent fraud in a more targeted manner.

## Theoretical analysis

### Definition of employee fraud

We adopt the definition of ACFE for employee fraud: the use of one’s occupation for personal enrichment through the deliberate misuse or misapplication of the employing organization’s resources or assets. This definition has three advantages^1^: First, the ACFE definition of fraud highlights that employees are the executors of corporate fraud, fitting the purpose of this paper. Second, because the ACFE definition of fraud has a broad scope, classifying fraud by nature into three categories: asset misappropriation, corruption, and financial statement fraud, the definition is conducive to a comprehensive consideration of the sources of the motives for fraud. Third, the ACFE definition of fraud has a broader practical basis and is more recognized by anti-fraud practitioners. Since the first fraud report was published in 1996, ACFE has published 12 fraud research reports, which have become a globally recognized authority on corporate fraud and a must-read for many anti-fraud professionals. Based on the results of years of research, ACFE’s definition of fraud is clearly actionable for further research.

In terms of the classification, fraud can be divided into asset misappropriation, corruption, and financial statement fraud according to its nature, and can also be divided into management fraud and ordinary-employee fraud according to the perpetrator’s position. The distinction between management fraud and ordinary-employee fraud is of great significance in the study of the employee fraud. Firstly, they are different in the scope. Management fraud can involve all types of fraud, while ordinary-employee fraud rarely involves financial statement fraud (Amiram et al., 2018; Veetikazhi et al., 2022). Secondly, they are different in the consequences. According to *Occupational Fraud 2022*, frauds committed by staff-level perpetrators in the number of perpetrators and the number of cases accounted for a greater proportion, while frauds committed by higher-level perpetrators typically take longer to detect and cause larger losses. In addition to causing more direct losses, such as money losses, management fraud also causes more indirect losses, such as inducing subordinates to fraud. According to the ethical leadership theory, unethical behaviors of leaders can also be learned and imitated by subordinates, which seriously damage the ethical climate of the organization (Brown et al., 2005; Brown and Treviño, 2006). Finally, they are different in the causes. Because the higher-level perpetrators often have the ability to evade or override controls that would otherwise detect fraud, the main factor leading to the management fraud is considered to be the willingness or attitude of the perpetrators (Holtfreter, 2005; Blickle et al., 2006), while the main factor leading to the ordinary-employee fraud is considered to be the supervision and control faced by the perpetrators (Belle and Cantarelli, 2017; Kuenzi et al., 2020). In summary, the classification of fraud by position is of great significance in the study of the employee fraud, especially in regression models that predict fraud.

Concepts that are closer to employee fraud are unethical behavior and white-collar crime. Unethical behavior is defined as any behavior by a member of an organization that violates the widely accepted ethical norms of society (Jones, 1991). Certain behaviors of employees such as theft, sabotage, lying to customers, and misrepresentation in financial reports are considered as unethical behaviors. However, other negative workplace behaviors of employees, such as coming in late and leaving early and neglecting work, are not considered as unethical behaviors because they do not necessarily violate widely accepted ethical norms of society (Kish-Gephart et al., 2010). The concept of white-collar crime is first introduced by Sutherland (1940) and refers to crimes committed by corporate executives (white-collar employees) to distinguish this type of crimes from street crimes and violent crimes. White-collar criminals mostly have high social and economic status and usually use their position to commit crimes such as false financial reporting, stock market manipulation, embezzlement, swindling, bribery, personal income tax evasion, and selling economic information (Sutherland, 1940, 1945).

While there are similarities between employee fraud and unethical behavior or white-collar crime, there are also differences. Both employee fraud and unethical behavior are violations of social ethics by employees, but the differences are: (1) Their motives are different. Unethical behavior is not necessarily self-interest, but also includes the violation of ethics for the benefit of the organization, such as unethical pro-organizational behavior (Chen et al., 2016; Xu et al., 2021), while employee fraud is for personal benefit to the detriment of the organization. (2) They are different in the victim. The victim of employee fraud is the owners of the enterprise, while some of unethical behaviors, such as sexual harassment and unethical behavior outside the workplace, do not necessarily harm the interest of enterprise’s owners. Both employee fraud and white-collar crime use their positions to intentionally harm the interests of the enterprise. However, the differences are: (1) Their executors are different. The executor of employee fraud includes all employees of the enterprise, while the executor of white-collar crime is limited to white-collar employees. (2) They are different in the severity of the consequences. Employee fraud is not all crime, but also includes the general violations of the lesser circumstances, while white-collar crime is a crime against criminal law.

From the above analysis, it can be seen that the extension of unethical behavior is the widest, followed by employee fraud, and white-collar crime is the narrowest. Moreover, unethical behavior includes employee fraud, and employee fraud includes white-collar crime. Therefore, as for the motives of white-collar crime, we can safely incorporate them as the motives of employee fraud, and as for the motives of unethical behavior, we need to choose the motives of behaviors in line with the definition of employee fraud. There are abundant researches on the motives of unethical behaviors and white-collar crimes at the individual level (Kish-Gephart et al., 2010; Dorminey et al., 2012), providing an excellent reference for item sources in the development of scale of employee fraud motive.

### Definition and theory of employee fraud motive

Motive is defined as a reason for doing something, especially one that is hidden or not obvious. Then, the motives of employee fraud refer to the reasons for employee fraud, especially the hidden or not obvious reasons. Motive has two measuring dimensions: quantity and intensity. We develop the scale of employee fraud motive, which fix the number of employee fraud motives through theoretical and data analysis, and form a special tool to measure the intensity of employee fraud motive. At that time, the higher the enterprise score measured by this scale, the higher the motive intensity of employee fraud, meaning that the enterprise will have more frauds.

In order to ensure that the scale of employee fraud motive does not omit important factors, we sort out the existing mainstream fraud motive theory. At present, the mainstream fraud motive theory includes fraud triangle theory, GONE theory and diamond theory. In recent years, scholars have begun to explore the motive of fraud from the perspective of behavioral psychology, such as the use of theory of planned behavior to explain the occurrence of fraud.

Fraud triangle theory is the most widely used fraud motivation theory at present (Dorminey et al., 2012; Raval, 2018). Cressey (1950) first proposes the fraud triangle theory and hypothesized that, for an act of fraud to occur, each of three criteria must be present: (1) the actor experiences a non-shareable financial problem, (2) the actor has an opportunity to violate a position of trust, and (3) the actor is able to adjust his self-perception such that he believes such a violation does not constitute criminal behavior. These three conditions are later summed up as pressure, opportunity and rationalization.

According to the GONE theory, the motives of fraud are composed of G (Greed), O (Opportunity), N (Need) and E (Exposure; Bologna et al., 1992). When employees are greedy and in desperate need of money, they will cheat whenever there are opportunities and it is assumed that they will not be discovered later. Compared with the fraud triangle theory, the GONE theory interprets pressure as need and rationalization as greed, and adds exposure factors.

According to diamond theory, in addition to pressure, opportunity and rationalization factors, motives for fraud should also include capability factors (Wolfe and Hermanson, 2004). Diamond theory refines the opportunity factor in the fraud triangle theory, arguing that employees will not fraud if they do not have the capability to take advantage of opportunities to perform and hide fraud. As described by (Wolfe and Hermanson, 2004), opportunity opens the door to fraud for employees, and pressure and rationalization bring employees closer to the door, but employees must be capable to walk through the door and cover it up.

The theory of planned behavior is a famous social psychology theory. The theory of planned behavior holds that behavioral intention is the most direct factor affecting behavior, and behavioral intention is influenced by attitude, subjective norm and perceived behavioral control (Ajzen, 1985, 1991). Attitude toward performing the behavior is a person’s general feeling of favorableness about performing that behavior. Subjective norm is the social pressure that individual perceives when deciding whether or not to perform a particular behavior. Perceived behavioral control refers to the perceived ease or difficulty of performing the behavior in question. The theory of planned behavior has been widely used in behavior research and has been proved to have high explanatory and predictive power for (un)ethical behavior (Yoon, 2011; Black et al., 2021; Wang et al., 2022). In the field of fraud motives research, Carpenter and Reimers (2005) find through experimental studies that both attitude and subjective norm have significant predictive power for corporate managers’ fraud intentions, while perceived behavioral control has little effect on the prediction of fraud intentions. Cohen et al. (2010) find through coding analysis of corporate fraud news reports that, compared with other factors in the theory of planned behavior, subjective norm is less common in the media.

The components of the above theories are different. However, since these theories all study fraud, the components of these theories have correspondence among the theories. Rationalization, pressure and opportunity in the fraud triangle theory correspond to attitude, subjective norm and perceived behavioral control in the theory of planned behavior, respectively. Exposure in the GOEN theory is a refinement of opportunity in the fraud triangle theory. Capability in diamond theory is also a refinement of opportunity in fraud triangle theory. The correspondence of the components of these theories is shown in Table 1.

**Table 1:** Comparison of components of fraud motivation theories.

Theories	Components			
Fraud triangle theory	Rationalization	Pressure	Opportunity	
GONE theory	Greed	Need	Opportunity	Exposure
Diamond theory	Rationalization	Pressure	Opportunity	Capability
Theory of planned behavior	Attitude	Subjective norm	Perceived behavioral control	

### Dimensions of employee fraud motive

We develop the scale of employee fraud motive based on theory of planned behavior rather than other theories. This is to combine the widely applied basis of fraud triangle theory with the theoretical basis of behavioral psychology. On the one hand, compared with other fraud motive theories, fraud triangle theory is more widely used in research and practice. On the other hand, the fraud triangle theory is more based on practical experience and lacks the basis of behavioral psychology, so it is not conducive to explaining and predicting fraud at the individual level. The theory of planned behavior has been proved to have a high explanatory and predictive power for behavior, and its components correspond well with the rationalization, pressure and opportunity of fraud triangle theory. Therefore, we develop the scale of employee fraud motive based on theory of planned behavior, and divide the employee fraud motive into three dimensions: attitude, subjective norm, and perceived behavioral control.

Theory of planned behavior also provides a theoretical basis for further refinement of attitude, subjective norm and perceived behavioral control. (1) Attitude can be divided into cognitive component and affective component (Crites et al., 1994). The cognitive component refers to the evaluative description of the attitude object, including understanding, belief, doubt, and approval or disapproval. The emotional component refers to the personal emotional experience of the attitude object, such as respect or contempt, sympathy or indifference, like or dislike, etc. (2) Subjective norm can be divided into personal norm, descriptive norm and injunctive norm (Cialdini et al., 1991). Personal norm is a self-based standard or expectation of behavior that comes from a person’s inner values and is enforced through expectations of self-promotion or self-deprecation. Descriptive norm prescribes what is (or is) right to do, guiding one’s behavior by perceiving the behavior of the majority: “If everyone is doing it, thinking about it, or believing it, it must be a wise thing to do.” Injunctive norm dictates what should be done to guide someone’s behavior by perceiving the majority’s approval or disapproval of that person’s behavior. (3) Perceived behavioral control can be divided into internal factors and external factors (Ajzen and Madden, 1986). Internal factors refer to personal quality factors, including the amount of information an individual has, as well as personal skills, abilities and emotions. External factors are situational factors outside the individual.

Accordingly, we can divide the scale of employee fraud motive into three first-level dimensions and seven second-level dimensions. The details are shown in Table 2.

**Table 2:** The dimensions of employee fraud motive.

First-level dimensions	Second-level dimensions
Attitude	Cognitive component
	Affective component
Subjective norm	Personal norm
	Descriptive norm
	Injunctive norm
Perceived behavioral control	External factor
	Internal factor

## Scale development and data collection

### Sources for generating items

Based on the theory of planned behavior, we incorporate the research findings on the motive of unethical behavior and white-collar crime as well as the practical experience of anti-fraud as the items of our new scale.

The research findings on the motive of unethical behavior come from the classic reviews (Treviño et al., 2006; Kish-Gephart et al., 2010; Li, 2022). Treviño et al. (2006) divide the motive of unethical behavior into consciousness, judgment and intention according to the ethical decision-making process. Kish-Gephart et al. (2010) divide the motive of unethical behavior into three aspects: individual characteristics, moral issue characteristics and organizational environment characteristics. Li (2022) conducts a bibliometric analysis to describe the characteristics and trends of unethical pro-organizational behavior research in business and management, and provide a systematically, transparently, and visually reviewed the landscape and development process of unethical pro-organizational behavior research.

The research findings on the motive of white-collar crime also come from the classic reviews (Coleman, 1987; Benson et al., 2009; Alalehto, 2018). Coleman (1987) proposes a theoretical framework to explain the causes of white-collar crime from two aspects: motivation and opportunity. Benson et al. (2009) summarize the core theories of environmental criminology: routine activity theory, crime pattern theory, and situational crime prevention theory, and believed that these three theories are applicable to white-collar crime and can be used to analyze opportunity structure. Alalehto (2018) reviews relevant researches on white-collar crime from the perspectives of agency logic and structural logic.

The practice experience of anti-fraud comes from *Fraud Auditing Standards*. The *Fraud Auditing Standards* uses the fraud triangle theory, which considers the three dimensions of fraud motive, including pressure, opportunity, and rationalization, and lists the precursor manifestations of perpetrators under each dimension.

### Item generation

Based on the above sources, we refined and generated 48 primary items. Among them, the attitude dimension contains nine indicators and 19 items, the subjective norm includes eight indicators and 17 items, and the perceived behavioral control includes eight indicators and 12 items. These items are specified in Table 3.

**Table 3:** The primary items of scale of employee fraud motive.

**1st-level dimensions**	**2nd-level dimensions**	**Indicators (sources)**	**Items**
Attitude	Cognitive component	Moral awareness (Reynolds, 2006)	Knowing that the fraud is wrong before exposure
		Cognitive moral development (Blasi, 1980)	Usually knowing right from wrong
		Moral philosophies (Henle et al., 2005)	Usually caring about the people around them
		Moral identity (Aquino and Reed, 2002)	Usually attaching great importance to moral cultivation
		Responsibility (Collins and Schmidt, 1993)	Usually being responsible
		Moral intensity (Jones, 1991; Paolillo and Vitell, 2002)	Claiming their frauds hurt no one; Claiming their frauds do not harm those they know well; Claiming their frauds are for good causes; Claiming their frauds are common actions; Claiming the adverse consequences of their frauds are not serious; Claiming the probability of adverse consequences from their frauds is very small; Claiming their frauds will have few adverse consequences in the near future
	Affective component	Moral emotion (Eisenberg, 2000)	Usually feeling no guilt or shame for their mistakes
		Locus of control (Trevino and Youngblood, 1990)	Usually tending to attribute it to external factors rather than to themselves
		Moral disengagement (Bandura, 1999)	Usually tending to make excuses for their mistakes; claiming the company owes them; Claiming they are just borrowing and will pay back later; Claiming they will pay the company more in other ways; Believing certain things, such as honor or integrity, are expendable
Subjective norm	Personal norm	Personal financial pressure (Agnew, 1992)	Trying to relieve their financial pressure by their frauds; Usually living beyond their means; Being unable to pay their debt before their frauds; Having bad credit histories before their frauds; Suffering from personal financial losses before their frauds; Encountering unexpected financial needs before their frauds; Usually engaging in bad behaviors such as gambling, drug abuse, alcoholism, visiting prostitutes and extramarital affairs; Claiming that once they get through their financial difficulties, they make up for the gaps created by their frauds
	Descriptive norm	Training (Weaver et al., 1999)	Usually lacking ethics training
		Family education (Demuth and Brown, 2004)	Lacking good family education
	Injunctive norm	Compensation incentives (Hill et al., 1992)	Usually being paid based on performance
		Performance goals (Schweitzer et al., 2004)	Trying to achieve performance goals through their frauds
		Job satisfaction (Judge et al., 2006)	Usually being dissatisfied with their jobs
		Work pressure (*Fraud Auditing Standards*)	Usually being not recognized for their performance; Usually being very concerned about losing their jobs; Usually being very eager to be promoted; Usually claiming they are being paid far less than they contribute
Perceived behavioral control	External factor	Power (Dunn, 2004)	Usually holding a great deal of power
		Economic temptation (Hegarty and Sims, 1978)	Profiting greatly from their frauds
	Internal factor	Greedy (*Fraud Auditing Standards*)	Usually being very greedy
		Self-control (Hirschi and Gottfredson, 1987)	Usually having good self-control
		Machiavellian (Hegarty and Sims, 1979; Dahling et al., 2009)	Usually being very utilitarian and only looking at the results, not the process; Usually having a strong desire to control; Usually having a strong desire for money, power and status; Usually distrusting others
		Self-efficacy (Flannery and May, 2000)	Usually being confident in their capabilities
		Hedonism (Blickle et al., 2006)	Usually liking to enjoy life
		Information asymmetry (Dunk, 1993)	Usually having more information at work that only they know; Usually performing work that is difficult to judge the quality of

### Data collection

We sent questionnaires online to 908 member companies of the Enterprise Anti-Fraud Alliance (EAFA)^2^ on 24 September, 2021. The contents of the questionnaire include the 48-item initial employee fraud motivation scale and the occurrence of enterprise fraud. Part of the questionnaire used in our study is described in the online Supplementary material. The scale is originally developed in English, we follow the “translation and back translation” procedure to translate it into Chinese. The questionnaire is filled by the person in charge of internal audit of the enterprise. Before filling out the scale, the questionnaire fillers read “Based on your experience, do most of the perpetrators found in your company in the past year fit the description below.” All items are scored on a 7-point scale ranging from 1 = *Strongly Disagree* to 7 = *Strongly Agree*. The questionnaires were collected on 17 November, 2021. A total of 514 questionnaires were collected, and 504 were finally valid. Among the 504 samples, there are 153 listed companies, accounting for 30.36%, and 351 unlisted companies, accounting for 69.64%. In that year, the number of sample enterprises with fraud was 371, accounting for 73.61%, and the number of sample enterprises without fraud was 133, accounting for 26.39%. Since only enterprises with fraud can fill out the employee fraud motive scale, we mainly analyzed 371 samples with fraud.

## Data analysis

We randomly divide the sample firms in which fraud occurred (sample size *N* = 371) into two groups with similar numbers and conducted exploratory factor analysis (*N* = 185) and confirmatory factor analysis (*N* = 186), respectively. Among them, the exploratory factor analysis helps us to filter out the items with high information content and generate the final employee fraud motive scale. The confirmatory factor analysis helps us to test the reliability and validity of the employee fraud motive scale. Subsequently, the regression results of fraud occurrence and scale score are used to verify the predictive validity of the scale. Finally, a mean score analysis is used to show the internal structure of employee fraud motive.

### Exploratory factor analysis

Before exploratory factor analysis, we need to perform preliminary tests to ensure that the sample is eligible for factor analysis. The KMO test shows that the KMO index of the sample is 0.806, which is higher than the standard of 0.7, indicating that we have an adequate sample size. Bartlett test of sphericity has value of *p* = 0.000 < 0.001, Chi-square = 1826.769, and degree of freedom = 91, indicating that our sample is suitable for factor analysis.

We conduct an exploratory factor analysis (principal axis factoring) with an oblique rotation (direct) oblimin allowing for correlations among factors. During the exploratory factor analysis, items with factor loadings less than 0.4 or multiple loadings on different factors are gradually removed. After repeated factor analysis and deletion of items, 14 items remain on the employee fraud motive scale, as shown in Table 4, and these items are distributed more evenly and appropriately across the three factors. The attitude factor contains five items. The subjective norm factor contains five items. The perceived behavioral control factor contains four items.

**Table 4:** The factor matrix of the employee fraud motive scale (*N* = 185).

**Items**	**Attitude**	**Subjective norm**	**Perceived behavioral control**
Claiming they will pay the company more in other ways	**0.580**	0.065	0.299
Believing certain things, such as honor or integrity, are expendable	**0.626**	0.011	0.333
Claiming the adverse consequences of their frauds are not serious	**0.770**	0.010	0.152
Claiming the probability of adverse consequences from their frauds is very small	**0.887**	0.088	0.027
Claiming their frauds will have few adverse consequences in the near future	**0.833**	0.042	0.047
Usually engaging in bad behaviors such as gambling, drug abuse, alcoholism, visiting prostitutes and extramarital affairs	0.089	**0.757**	0.124
Usually being dissatisfied with their jobs	0.035	**0.884**	0.006
Usually being very eager to be promoted	0.031	**0.805**	0.070
Claiming that once they get through their financial difficulties, they make up for the gaps created by their frauds	0.005	**0.872**	0.026
Usually living beyond their means	0.080	**0.773**	0.058
Usually liking to enjoy life	0.205	−0.050	**0.640**
Usually being very utilitarian and only looking at the results, not the process	0.053	0.082	**0.656**
Usually holding a great deal of power	0.091	0.065	**0.771**
Usually being very greedy	0.156	0.136	**0.772**

Statistics that load ≥ 0.40 are in bold.

### Confirmatory factor analysis

On the basis of exploratory factor analysis, we perform confirmatory factor analysis on another group of samples (*N* = 186). Confirmatory factor analysis mainly examines the discriminant validity, convergent validity and reliability of the employee fraud motive scale.

#### Discriminant validity

First, we set up two competing alternative models (two-factor model and one-factor model) to compare with the basic three-factor model to determine the optimal model. The results of the comparison of the goodness of fit metrics of the three models (Table 5) show that the three-factor model has the best fit. Specifically, the *χ*^2^/*df* of the three-factor model is 4.722, the RMSEA is 0.141, and the SRMR is 0.061, all of which are smaller than the values of the other two models. The CFI of the three-factor model is 0.847 and the TLI is 0.812, and these values are larger than the values of the other two models. In summary, it is clear that the basic model is better than the other alternative models and the structure of the three-factor model is validated. The results support the discriminant validity of the scale.

**Table 5:** The goodness of fit metrics for the three candidate models (*N* = 186).

Models	*χ* ^2^	*df*	*χ*^2^/*df*	RMSEA	SRMR	CFI	TLI
Three-factor model	349.401	74	4.722	0.141	0.061	0.847	0.812
Two-factor model	558.196	75	7.443	0.186	0.109	0.732	0.675
One-factor model	1112.835	77	14.452	0.269	0.247	0.425	0.321

The three-factor model is a model in which attitude, subjective norm, and perceived behavioral control are each one factor. The two-factor model is a model in which attitude and perceived behavioral control are combined as one factor and subjective norm is one factor; the one-factor model is a model in which attitude, subjective norm, and perceived behavioral control are combined as one factor.

Second, we compare the square root of the average variable extracted (AVE) for each dimension of employee fraud motive with the correlation coefficient between the dimensions (Table 6). The square root of AVE for attitude is 0.789, the square root of AVE for perceived behavioral control is 0.759, and the square root of AVE for subjective norm is 0.840. They are all greater than the correlation coefficients of the rows and columns in which they are located. These results also support the discriminant validity of the scale.

**Table 6:** Correlation coefficient matrix between dimensions and AVE of each dimension.

	**Attitude**	**Subjective norm**	**Perceived behavioral control**
Attitude	(0.789)		
Subjective norm	0.392	(0.759)	
Perceived behavioral control	0.145	0.186	(0.840)

The value in parentheses is the square root of the AVE of the dimension.

#### Convergent validity and reliability

Table 7 reports the results of the tests of the convergent validity and reliability of the employee fraud motive scale. In terms of convergent validity, the AVE of the scale and the AVE of each subscale exceed 0.5, supporting the convergent validity of the scale. In terms of reliability, the internal consistency (*α*) for the scale and the *α* for each subscale exceed 0.8, and the composite reliability (*γ*) for the scale and the *γ* for each subscale exceed 0.7, supporting the reliability of the scale.

**Table 7:** The convergent validity and reliability of the scale.

	**AVE**	***α***	***γ***
Employee fraud motive	0.607	0.871	0.955
Attitude	0.623	0.885	0.892
Subjective norm	0.706	0.920	0.923
Perceived behavioral control	0.577	0.848	0.845

### Predictive validity

The results so far are necessary but not sufficient to demonstrate the utility of the new measure of employee fraud motives. Predictive validity also has to be established in the construct validation process. We match the questionnaire data with the corporate basic information data and finally obtain 298 valid samples. Using these samples, we run linear regressions of the amount of fraud losses (*Loss*), the total duration of frauds (*Duration*), the number of frauds (*Frauds*), and the number of perpetrators (*Perpetrators*) on the score of employee fraud motive (*Motive*). In the regression model, the control variables include registered capital (*Capital*), number of employees (*Employees*), firm age (*Firm_age*), whether the firm is listed or not (*List*), the shareholding ratio of the first largest shareholder (*Top1*), whether the firm is registered in one of the developed provinces^3^ (*Developed*), whether the fraud involves the management (*Management*), and industry fixed effects (*Industry*). See Table 8 for the variable definitions. Among them, the data on the occurrence of corporate fraud and the score of employee fraud motive are obtained from the questionnaire survey, and the data on the control variables are obtained from the data of corporate basic information.^4^

**Table 8:** Definitions of the variables in predictive validity test.

**Variables**	**Definitions**
*Loss*	The natural logarithm of the total amount of fraud losses
*Duration*	The natural logarithm of the total number of months all frauds lasted
*Frauds*	The number of frauds
*Perpetrators*	The number of perpetrators
*Motive*	The mean score of the 14 items in the employee fraud motive scale
*Capital*	The natural logarithm of the registered capital
*Employees*	The natural logarithm of the number of employees
*Firm_age*	The natural logarithm of the firm age
*List*	The dummy variable if the firm is listed, equals 1, otherwise equals 0
*Top1*	The shareholding ratio of the first largest shareholder
*Developed*	The dummy variable if the firm is registered in one of the developed provinces (Beijing, Shanghai, Guangdong, Jiangsu and Zhejiang), equals 1, otherwise equals 0
*Management*	The dummy variable if the fraud involves the management, equals 1, otherwise equals 0
*Industry*	Industry fixed effect. The industry classification is based on the 2012 industry classification of the China Securities Regulatory Commission

To ensure that the sample of predictive validity is appropriate, descriptive statistics have been performed on variables participating in the regression to test for the presence of outliers in these samples. The descriptive statistics (Table 9) show that the values of all variables are in the normal range, indicating that the sample is suitable for predictive validity. The mean of *Loss* is 14.567, suggesting that a firm suffers a loss of 2.120 million (e^14.567^) from fraud on average. The mean of *Duration* is 3.985, suggesting that in a firm all frauds in total last 53.785 (e^3.985^) months on average. The mean of *Frauds* is 8.698, suggesting that a firm discovers approximately 8.698 fraud cases on average. The mean of *Perpetrators* is 14.205, suggesting that a firm discovers approximately 14.205 perpetrators on average. The mean of *Motive* is 4.219, indicating that the employee fraud motive in the sample tends to show a right-leaning normal distribution.

**Table 9:** Descriptive statistics of the variables in predictive validity test.

**Variables**	***N***	**Mean**	**SD**	**Min**	**Median**	**Max**
*Loss*	298	14.567	1.716	12.429	14.914	17.823
*Duration*	298	3.985	1.315	1.099	4.094	6.805
*Frauds*	298	8.698	10.096	1.000	5.000	41.000
*Perpetrators*	298	14.205	17.104	0.000	8.000	68.000
*Motive*	298	4.219	0.716	1.000	4.208	6.433
*Capital*	298	10.592	1.728	4.605	10.820	14.145
*Employees*	298	8.055	1.382	3.434	8.006	12.899
*Firm_age*	298	2.610	0.660	0.693	2.773	3.689
*List*	298	0.332	0.472	0.000	0.000	1.000
*Top1*	298	0.674	0.304	0.078	0.716	1.000
*Developed*	298	0.614	0.488	0.000	1.000	1.000
*Management*	298	0.336	0.473	0.000	0.000	1.000

The regression results (Table 10) show that the regression coefficients of employee fraud motive are significantly positive, indicating that employee fraud motivate is positively related to the amount of fraud occurring in the firm. As shown in column (1), the coefficient of *Loss* on *Motive* is significantly positive at 1% level, which shows that the employee fraud motive significantly increases the fraud losses. As shown in column (2), the coefficient of *Duration* is significantly positive at 1% level, which shows that the employee fraud motive significantly increases the duration of frauds. As shown in column (3), the coefficient of *Frauds* is significantly positive at 1% level, which shows that the employee fraud motive significantly increases the number of frauds. As shown in column (4), the coefficient of *Perpetrators* is significantly positive at 1% level, which shows that the employee fraud motive significantly increases the number of perpetrators. Overall, the results support the predictive validity of the scale.

**Table 10:** Predictive validity test of the employee fraud motive scale.

	(1)	(2)	(3)	(4)
	*Loss*	*Duration*	*Frauds*	*Perpetrators*
*Motive*	0.386^***^	0.367^***^	1.870^***^	3.241^***^
	(0.147)	(0.107)	(0.680)	(1.159)
*Capital*	0.073	0.017	−0.039	−0.434
	(0.065)	(0.045)	(0.373)	(0.705)
*Employees*	0.269^***^	0.272^***^	1.953^***^	3.856^***^
	(0.073)	(0.061)	(0.621)	(1.030)
*Firm_age*	−0.080	−0.048	−1.767^**^	−1.135
	(0.165)	(0.117)	(0.866)	(1.484)
*List*	0.207	−0.543^***^	−2.782^**^	−3.340
	(0.224)	(0.169)	(1.351)	(2.269)
*Top1*	−0.290	−0.815^***^	−4.097^*^	−0.649
	(0.337)	(0.270)	(2.215)	(3.752)
*Developed*	0.258	0.007	0.434	−1.583
	(0.190)	(0.142)	(1.152)	(2.145)
*Management*	0.873^***^	0.545^***^	2.613^**^	3.498
	(0.203)	(0.150)	(1.275)	(2.240)
*Constant*	9.717^***^	0.972	−5.496	−20.184^*^
	(0.955)	(0.748)	(6.234)	(11.679)
*Industry*	Yes	Yes	Yes	Yes
*R* ^2^	0.199	0.226	0.157	0.137
Adjusted *R*^2^	0.163	0.191	0.119	0.098
*F*	5.961	6.767	2.995	3.338
*N*	298	298	298	298

Robust standard errors are in parentheses. ^***^, ^**^, and ^*^ indicate 1%, 5%, and 10% significance levels, respectively.

### Internal structure of employee fraud motive

So far, our results have verified the reliability and validity of the employee fraud motive scale. The scale can be used not only to explain and predict the amount of fraud in enterprises, but also to analyze the internal structure of employee fraud motive. Using the whole sample of questionnaires (*N* = 371), the analysis results (Table 11) show that the factor with the highest mean scores is perceived behavioral control, followed by attitude, and finally subjective norm. It also means that the most important factor to constitute the motive of fraud is the perceived behavioral control, followed by attitude, and finally subjective norm.

**Table 11:** Internal structure of employee fraud motive.

**Items**	**Mean score**
Claiming they will pay the company more in other ways	4.426
Believing certain things, such as honor or integrity, are expendable	4.566
Claiming the adverse consequences of their frauds are not serious	4.695
Claiming the probability of adverse consequences from their frauds is very small	4.720
Claiming their frauds will have few adverse consequences in the near future	4.617
Attitude	**4.605**
Usually engaging in bad behaviors such as gambling, drug abuse, alcoholism, visiting prostitutes and extramarital affairs	3.523
Usually being dissatisfied with their jobs	3.313
Usually being very eager to be promoted	3.164
Claiming that once they get through their financial difficulties, they make up for the gaps created by their frauds	3.108
Usually living beyond their means	3.345
Subjective norm	**3.291**
Usually liking to enjoy life	5.005
Usually being very utilitarian and only looking at the results, not the process	4.884
Usually holding a great deal of power	4.693
Usually being very greedy	4.903
Perceived behavioral control	**4.871**
Employee fraud motive	**4.256**

The bold values are aggregated statistics.

## Conclusion and discussion

Based on the theory of planned behavior, our study integrates the existing research findings on driving factors of employee fraud and anti-fraud practical experience, extracts the key factors of employee fraud motive, and develops a multidimensional scale of employee fraud motive. The exploratory factor analysis generates three subscales, comprising 14 items, measuring attitude, subjective norm and perceived behavioral control of employee fraud motive. The confirmatory factor analysis supports the reliability, discriminant validity and convergent validity of the new scale. The multiple regression results show that the score of employee fraud motive is positively correlated with the amount of employee fraud occurrence, indicating that the predictive validity of the scale holds. Overall, the scale developed in our study displays good reliability and validity, and is worth spreading.

The main contributions of our research are as follows. Firstly, based on the behavioral perspective, we summarize the fraud motives at the employee level and develop the employee fraud motive scale, enriching the research on fraud motives. Secondly, our study theoretically and quantitatively integrates the existing research findings and practice experience to identify the key factors of fraud motive, which not only implicate researchers to focus on the important issues, but also help anti-fraud practitioners to save costs. Thirdly, our research comprehensively and directly measures the employee fraud motive by developing a scale, providing a quantitative basis for empirical research on employee fraud motive. Finally, our study provides an operational tool for corporate anti-fraud practitioners to diagnose the causes of fraud and to prevent fraud in a more targeted manner.

Our study also has the following practical implications: First, existing research findings and practical experience have identified numerous fraud motives, but not all of them are important. The items in the employee fraud motive scale developed in our study are the key factors of fraud motive, and anti-fraud practitioners can save a lot of cost by referring to these key factors to identify and prevent fraud. Secondly, this study finds that the most important factor that constitutes fraud motives is perceived behavioral control, followed by behavioral attitudes and finally subjective norm. Therefore, when enterprises are faced with anti-fraud resource investment constraints, they can also allocate resources following this order of priority. Finally, enterprises can establish a personal fraud risk assessment and early warning system for employees to manage fraud risk and focus on monitoring employees with higher fraud risk. For example, employees who usually like to enjoy life, are utilitarian, hold greater power, and are greedy have higher fraud risks. For these employees, companies need to focus on prevention.

Our study has limitations that provide promising directions for future research. First, in order to implicate researchers to focus on the important issues and help anti-fraud practitioners to save costs, we only extract the key factors instead of all factors of employee fraud motive to develop the employee fraud motive scale. Even though we integrate the existing research findings and practice experience about the motives of fraud and verify the reliability and validity of the scale, it is difficult to ensure that no other key factors are missed. Therefore, future research should further examine whether the scale misses key factors. Second, due to the limitations of available data, in the predictive validity test, we only consider the classification of fraud by the perpetrator’s position, rather than the usual classification of fraud types, such as asset misappropriation, corruption, and financial statement fraud. Future research should collect richer data to test the predictive validity of the scale for the three different types of fraud. Finally, we use a sample of Chinese companies to test the reliability and validity of the scale. China is the world’s most populous country and the world’s second largest economy, so the scale has a wide range of applications. However, China is still an emerging market country with unique institutions and culture, so the scale is not necessarily applicable to other countries. Future research should be conducted across cultures to test the applicability of the scale.

## Data availability statement

The data analyzed in this study is subject to the following licenses/restrictions: The data presented in this paper are available on request from the corresponding author. Requests to access these datasets should be directed to huangjq65@mail2.sysu.edu.cn.

## Author contributions

BL: conception, funding, data collection, and advice. JH and YL: conception, writing manuscript, and data analysis. SL and HZ: revising manuscript and advice. All authors contributed to the article and approved the submitted version.

## Funding

This research was funded by the National Social Science Foundation of China (grant number 18VSJ082), Philosophy and Social Science Foundation of Guangdong Province (grant number GD21TW07-01), Famous Accounting Training Project of China Ministry of Finance (grant number [2016] No.15), and Science and Technology Plan Project of Guangzhou (grant number 202200110001).

## Conflict of interest

The authors declare that the research was conducted in the absence of any commercial or financial relationships that could be construed as a potential conflict of interest.

## Publisher’s note

All claims expressed in this article are solely those of the authors and do not necessarily represent those of their affiliated organizations, or those of the publisher, the editors and the reviewers. Any product that may be evaluated in this article, or claim that may be made by its manufacturer, is not guaranteed or endorsed by the publisher.

## Supplementary material

The Supplementary material for this article can be found online at: https://www.frontiersin.org/articless/10.3389/fpsyg.2022.1026519/full#supplementary-material

## Footnotes

^1^Not all definitions of fraud have these advantages. For example, U.S. *National Auditing Standard (NAS) Circular 82* defines fraud as the intentional misstatement and omission of financial reporting by a company or enterprise. Under this definition, the executor of fraud is the company or enterprise, and the nature of fraud is limited to financial reporting practices only. Another example is the IIA’s definition of fraud: an act in which the perpetrator intentionally deceives and damages others for his or her own personal benefit. Although it is close to ACFE, the public practice basis is insufficient.

^2^The Enterprise Anti-Fraud Alliance (EAFA), formerly known as the China Enterprise Anti-Fraud Alliance, was founded in 2015 by the Guangdong Provincial Enterprise Internal Control Association, China Vanke, Alibaba, Shimao, CIMC, Midea, Country Garden, Fosun and the Internal Control Research Center of Enterprises and non-profit organizations of Sun Yat-sen University. The EAFA is committed to corporate anti-fraud research and standard setting, perpetrator blacklist sharing, mutual assistance of anti-fraud resources, and certification of international Certified Anti-Fraud Professional (CAP), providing Chinese wisdom and Chinese solutions for the global anti-fraud cause.

^3^Following Chen et al. (2022), the developed provinces we define include Beijing, Shanghai, Guangdong, Jiangsu and Zhejiang.

^4^The data of corporate basic information is manually collected from *Qichacha* (https://www.qcc.com/), one of the largest enterprise credit information inquiry platforms in China.
