# Supplementary material for: Why do employees commit fraud? Theory, measurement, and validation
Source: Front Psychol. 2022 Oct 6;13:1026519. doi: 10.3389/fpsyg.2022.1026519 (PMC9590450; doi:10.3389/fpsyg.2022.1026519)
Supplement: Supplementary file 1 [file Data_Sheet_1.docx]

Supplementary Material

# Part of the questionnaire used in the study

**1.1 Has your company detected any fraud cases in 2020?**

A. Yes (Please fill in the number of cases: __) B. No

**1.2 Among the fraud cases discovered by your company in 2020, what is the average duration of fraud (i.e. the time distance from the beginning of fraud to the time when fraud is discovered)?**

| A. Less than 6 months | B. 7-12 months |
| --- | --- |
| C. 13-18 months | D. 19-24 months |
| E. 25-36 months | F. 37-48 months |
| G. 49-60 months | H. Over 60 months |

**1.3 What is the amount of money your company is likely to lose in fraud cases found by your company in 2020?**

| A. Less than RMB 500,000 | B. 500,000 yuan to less than 1 million yuan |
| --- | --- |
| C. 1 million yuan to less than 5 million yuan | D. 5 million yuan to less than 10 million yuan |
| E. 10 million yuan to less than 20 million yuan | F. 20 million yuan to less than 30 million yuan |
| G. 30 million yuan to less than 40 million yuan | H.40 million yuan to less than 40 million yuan |
| I. 50 million yuan and above |  |

**1.4 what is the number of perpetrators your company found in each department in 2020?**

| 1. Production or operations_____ | 1. Accounting_____ |
| --- | --- |
| 1. Sales _____ | 1. Customer Service_____ |
| 1. Purchasing _____ | 1. Finance_____ |
| 1. Human Resources_____ 2. R&D_____ | 1. Management_____ 2. Warehousing and Logistics_____ |
| 1. IT _____ | L. Others (please fill in department__)____ |

**1.5 Based on your experience, do most of the perpetrators found in your company in 2020 fit the description below?**

| Items | \| Strongly Disagree———————Strongly Agree \| \| \| \| \| \| \| \| --- \| --- \| --- \| --- \| --- \| --- \| --- \| \| 1 \| 2 \| 3 \| 4 \| 5 \| 6 \| 7 \| |
| --- | --- | --- | --- | --- | --- | --- | --- | --- | --- | --- | --- | --- | --- | --- | --- |
| Knowing that the fraud is wrong before exposure. |  |
| Usually knowing right from wrong. |  |
| Usually caring about the people around them. |  |
| Usually attaching great importance to moral cultivation. |  |
| Usually being responsible. |  |
| Claiming their frauds hurt no one;  Claiming their frauds do not harm those they know well;  Claiming their frauds are for good causes;  Claiming their frauds are common actions;  Claiming the adverse consequences of their frauds are not serious;  Claiming the probability of adverse consequences from their frauds is very small;  Claiming their frauds will have few adverse consequences in the near future. |  |
| Usually feeling no guilt or shame for their mistakes. |  |
| Usually tending to attribute it to external factors rather than to themselves. |  |
| Usually tending to make excuses for their mistakes; claiming the company owes them;  Claiming they are just borrowing and will pay back later;  Claiming they will pay the company more in other ways;  Believing certain things, such as honor or integrity, are expendable. |  |
| Trying to relieve their financial pressure by their frauds;  Usually living beyond their means;  Being unable to pay their debt before their frauds;  Having bad credit histories before their frauds;  Suffering from personal financial losses before their frauds;  Encountering unexpected financial needs before their frauds;  Usually engaging in bad behaviors such as gambling, drug abuse, alcoholism, visiting prostitutes and extramarital affairs;  Claiming that once they get through their financial difficulties, they make up for the gaps created by their frauds. |  |
| Usually lacking ethics training. |  |
| Lacking good family education. |  |
| Usually being paid based on performance. |  |
| Trying to achieve performance goals through their frauds. |  |
| Usually being dissatisfied with their jobs. |  |
| Usually being not recognized for their performance;  Usually being very concerned about losing their jobs;  Usually being very eager to be promoted;  Usually claiming they are being paid far less than they contribute. |  |
| Usually holding a great deal of power. |  |
| Profiting greatly from their frauds. |  |
| Usually being very greedy. |  |
| Usually having good self-control. |  |
| Usually being very utilitarian and only looking at the results, not the process;  Usually having a strong desire to control;  Usually having a strong desire for money, power and status;  Usually distrusting others. |  |
| Usually being confident in their capabilities. |  |
| Usually liking to enjoy life. |  |
| Usually having more information at work that only they know;  Usually performing work that is difficult to judge the quality of. |  |
